# Predictive Models Based on Molecular Images and Molecular Descriptors for Drug Screening

**DOI:** 10.1021/acsomega.3c04073

**Published:** 2023-09-13

**Authors:** Hideaki Mamada, Mari Takahashi, Mizuki Ogino, Yukihiro Nomura, Yoshihiro Uesawa

**Affiliations:** † Drug Metabolism and Pharmacokinetics Research Laboratories, Central Pharmaceutical Research Institute, Japan Tobacco Inc., 1-1 Murasaki-cho, Takatsuki, Osaka 569-1125, Japan; ‡ Department of Medical Molecular Informatics, Meiji Pharmaceutical University, 2-522-1 Noshio, Kiyose, Tokyo 204-858, Japan

## Abstract

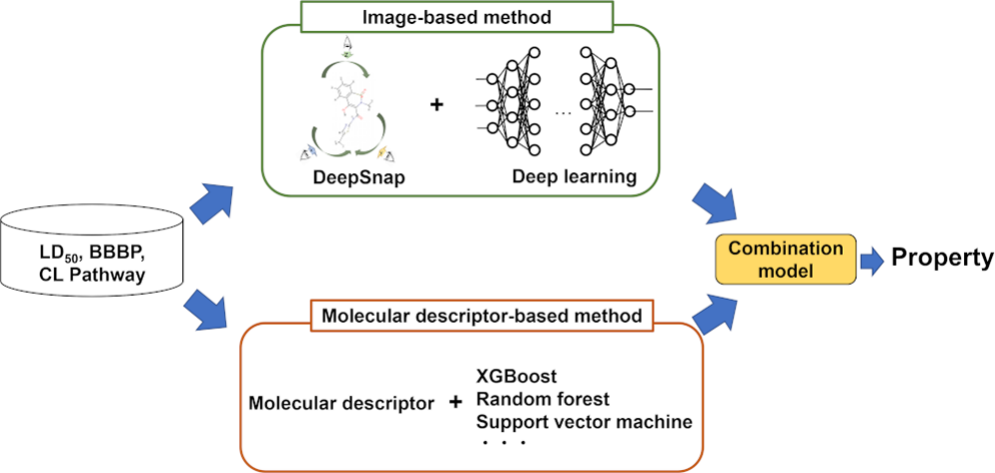

Various toxicity and pharmacokinetic evaluations as screening experiments are needed at the drug discovery stage. Currently, to reduce the use of animal experiments and developmental expenses, the development of high-performance predictive models based on quantitative structure–activity relationship analysis is desired. From these evaluation targets, we selected 50% lethal dose (LD_50_), blood–brain barrier penetration (BBBP), and the clearance (CL) pathway for this investigation and constructed predictive models for each target using 636–11,886 compounds. First, we constructed predictive models using the DeepSnap-deep learning (DL) method and images of compounds as features. The calculated area under the curve (AUC) and balanced accuracy (BAC) were, respectively, 0.887 and 0.818 for LD_50_, 0.893 and 0.824 for BBBP, and 0.883 and 0.763 for the CL pathway. Next, molecular descriptors (MDs) of compounds were calculated using Molecular Operating Environment, alvaDesc, and ADMET Predictor to construct predictive models using the MD-based method. Using these MDs, we constructed predictive models using DataRobot. The calculated AUC and BAC were, respectively, 0.931 and 0.805 for LD_50_, 0.919 and 0.849 for BBBP, and 0.900 and 0.807 for the CL pathway. In this investigation, we constructed predictive models combining the DeepSnap-DL and MD-based methods. In ensemble models using the mean predictive probability of the DeepSnap-DL and MD-based methods, the calculated AUC and BAC were, respectively, 0.942 and 0.842 for LD_50_, 0.936 and 0.853 for BBBP, and 0.908 and 0.832 for the CL pathway, with improved predictive performance observed for all variables compared with either single method alone. Moreover, in consensus models that adopted only compounds for which the results of the two methods agreed, the calculated BAC for LD_50_, BBBP, and the CL pathway were 0.916, 0.918, and 0.847, respectively, indicating higher predictive performance than the ensemble models for all three variables. The predictive models combining the DeepSnap-DL and MD-based methods displayed high predictive performance for LD_50_, BBBP, and the CL pathway. Therefore, the application of this approach to prediction targets in various drug discovery screenings is expected to accelerate drug discovery.

## Introduction

To reduce the use of animal experiments and developmental expenses, predictive models based on quantitative structure–activity relationship (QSAR) analysis have been actively used in recent years at the drug discovery stage.^1−3^ QSAR analysis is an approach based on features of compounds such as molecular descriptors (MDs) and fingerprints, and algorithms such as support vector machine, random forest, artificial neural network, k-nearest neighbor, XGBoost, and deep learning (DL) have been applied.^1−4^ At the drug discovery stage, QSAR analysis is used to predict pharmacological activity, pharmacokinetic parameters, and toxicity parameters. Several predictive models based on QSAR analysis were previously reported for parameters associated with toxicity, such as inhibition of the human ether-a-go-go related gene and 50% lethal dose (LD_50_), and parameters associated with pharmacokinetics, such as metabolic stability, protein binding, distribution to blood cells, membrane permeability, clearance (CL), and the CL pathway.^5−11^ Therefore, these predictive models are used by various pharmaceutical companies to perform virtual screening, narrow hit compounds, and prioritize various experiments.^1−3,12^ Thus, predictive models have been reported for many toxicity parameters and pharmacokinetic parameters.^1−3,12^ However, for some targets, the predictive performance of these models is apparently insufficient. As an approach to improve the predictive performance for targets such as LD_50_, a consensus model that combines predictive models constructed by 35 different organizations was reported.^11^ As another approach to improve predictive performance, Li et al. proposed an approach constructing predictive models using multiple features of compounds (fingerprints and MDs), algorithms (support vector machine, random forest, k-nearest neighbor, and XGBoost), and endpoints (regression, multiclass, and binary).^13^ However, these approaches used 35 or more different predictive models for one evaluation target, and descriptors and algorithms need to be selected for each evaluation target. Thus, much effort is exhausted in constructing such models. Therefore, it is difficult to employ these approaches to construct predictive models at the drug discovery stage because there is more than one prediction target, and predictive models are updated whenever new data are obtained. Thus, approaches to improve predictive performance that can be easily applied to various evaluation targets are desired.

Recently, Uesawa developed the DeepSnap-DL method that constructs predictive models by DL using images of compounds as features.^14^ They reported that this approach had better predictive performance for toxicity parameters such as the activity of constitutive androstane receptor and aryl hydrocarbon receptor, mitochondrial membrane potential, and CL as the pharmaceutical target compared with conventional machine learning.^14−17^ Moreover, among these parameters, they reported an approach to improve the predictive performance of a classification model for CL that combines the DeepSnap-DL and MD-based methods.^15^ However, the use of this approach has been limited to the analysis of CL.

Thus, in this study, approaches aiming to improve the predictive performance of classifications using the DeepSnap-DL and MD-based methods were investigated for evaluation targets at the drug discovery stage. To construct these predictive models, we selected LD_50_ as the toxicity evaluation target and blood–brain barrier penetration (BBBP) and the CL pathway as the pharmacokinetic targets using publicly known data sets. LD_50_ is defined as the dose of a compound that can kill 50% of animals. Because compounds that exert their effects on the central nervous system must pass through the BBB, it is desired that their BBBP values are high.^18^ Moreover, the CL pathway is based on excretion routes, and CL pathways are classified mainly as hepatic metabolism and renal elimination. These CL pathways are important parameters for selecting the prediction method when predicting human CL.^19^ This series of parameters can be verified using animal experiments. However, such experiments are extremely expensive, and a long time is required to obtain experimental results. Thus, it is desirable to predict these parameters using QSAR analysis before synthesizing the compounds. Therefore, in this study, we applied predictive models combining the DeepSnap-DL and MD-based methods during drug discovery to improve the predictive performance of these evaluation targets.

## Results

### Splitting Data Sets into Training and Test Data Sets and Their Verification by Chemical Space Analysis

**Figure 1:**
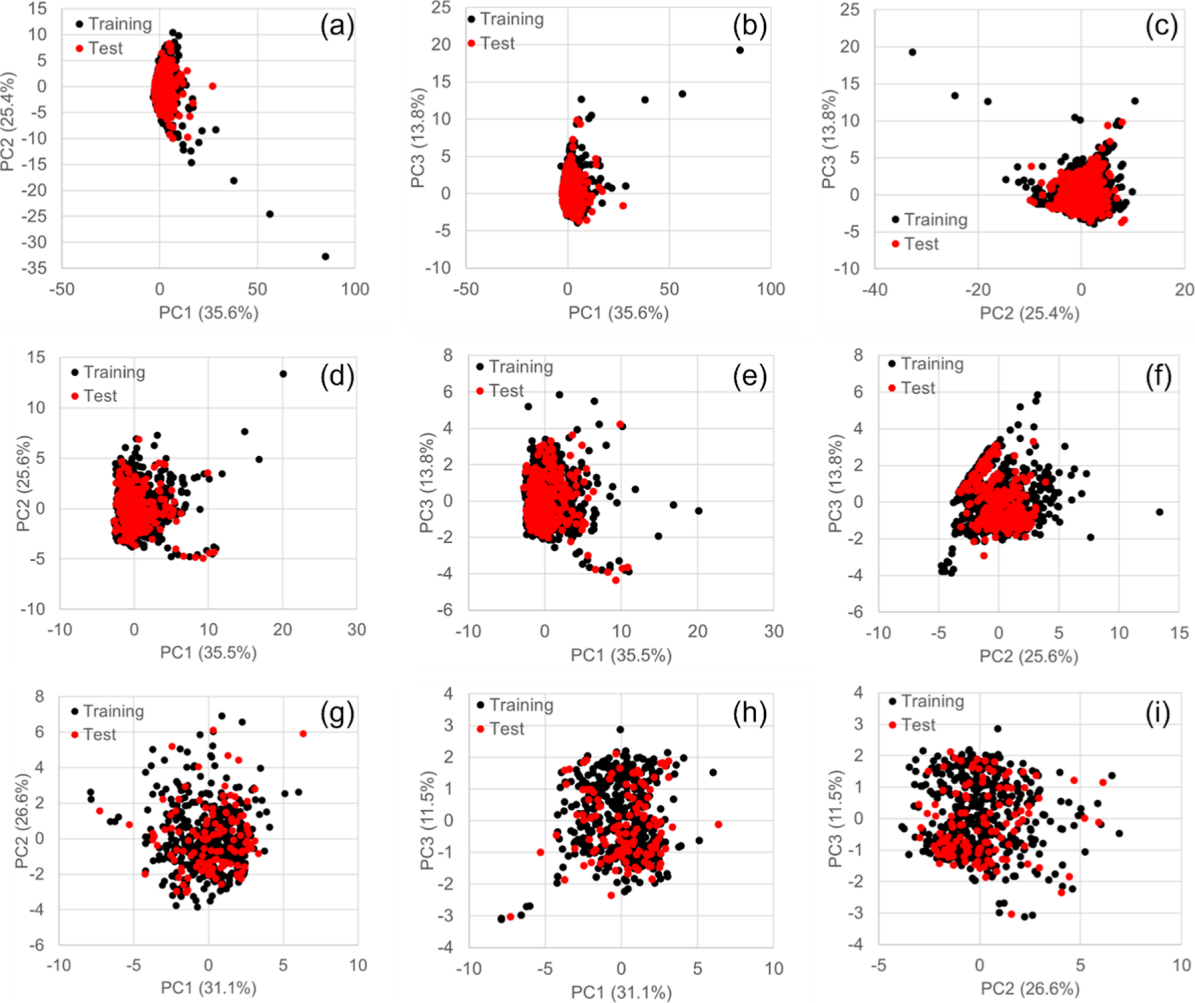
Eleven representative MD-based three-component PCA score plots for LD_50_ (*n* = 11,886), BBBP (*n* = 2049), and CL pathway prediction. (a,d,g) Score plot of PC1 and PC2. The horizontal and vertical axes represent PC1 and PC2, respectively. (b,e,h) Score plot of PC1 and PC3. The horizontal and vertical axes represent PC1 and PC3, respectively. (c,f,i) Score plot of PC2 and PC3. The horizontal and vertical axes represent PC2 and PC3, respectively. Each compound is indicated by a circle. Black circles represent training set compounds for LD_50_ (*n* = 8992), BBBP (*n* = 2049), and the CL pathway (*n* = 509), whereas red circles represent test set compounds for LD_50_ (*n* = 2894), BBBP (*n* = 409), and the CL pathway (*n* = 127). PCA, principal component analysis; LD_50_, 50% lethal dose; BBBP, blood–brain barrier penetration; CL, clearance; PC, principal component.

To confirm the correctness of the compound separation, principal component analysis (PCA) was performed using the data sets for LD_50_ (11,886 compounds), BBBP (2049 compounds), and the CL pathway (636 compounds) with 11 representative MDs. A previous study demonstrated that PCA could reveal an applicability domain.^20^ The variances of principal component (PC)1, PC2, and PC3 were 35.6, 25.4, and 13.8%, respectively, for LD_50_; 35.5, 25.6, and 13.8%, respectively, for BBBP; and 31.1, 26.6, and 11.5%, respectively, for the CL pathway. Effectively separated compounds into training and test data sets are presented in Figure 1.

### Construction of DeepSnap-DL Models for LD_50_, BBBP, and the CL Pathway

Previous studies examined multiple angles for the DeepSnap-DL method.^15^ For screening in drug discovery, the model was constructed using an angle of 145° for all three axes. Furthermore, the present study was conducted using five learning rates (10^–7^ to 10^–3^) and five maximum epochs (15–300). In each condition, the epoch with the lowest loss (DeepSnap [validation]) was selected as the epoch to calculate the evaluation metrics, and the model with the highest area under the curve (AUC) (DeepSnap [validation]) was selected as the final model in the DeepSnap-DL method. The highest AUC (DeepSnap [validation]) was determined for LD_50_ with a max epoch of 60 and a learning rate of 10^–5^, BBBP with a max epoch of 300 and a learning rate of 10^–4^, and the CL pathway with a max epoch of 15 and a learning rate of 10^–4^ (Table 1). At a learning rate of 10^–4^, the AUCs (DeepSnap [validation]) for BBBP for epochs of 30 and 300 were 0.9269 and 0.9271, respectively. The results of the test data sets in the final model in the DeepSnap-DL method calculated using these conditions are presented in Table 2. The AUC and balanced accuracy (BAC) were 0.887 and 0.818, respectively, for LD_50_, 0.893 and 0.824, respectively, for BBBP, and 0.883 and 0.763, respectively, for the CL pathway. The results for LD_50_, BBBP, and the CL pathway at each seed are shown in Tables S1–S3.

**Table 1:** AUCs of the Validation Results Using the DeepSnap-DL Method^a^

			max epoch				
			15	30	60	100	300
LD_50_	learning rate	10^–3^	0.500	0.500	0.500	0.500	0.500
		10^–4^	0.853	0.816	0.827	0.853	0.837
		10^–5^	0.828	0.860	**0.866**	0.864	0.863
		10^–6^	0.726	0.746	0.771	0.802	0.841
		10^–7^	0.622	0.645	0.721	0.725	0.748
BBBP		10^–3^	0.911	0.907	0.903	0.904	0.923
		10^–4^	0.924	0.927	0.926	0.924	**0.927**
		10^–5^	0.871	0.877	0.907	0.917	0.917
		10^–6^	0.808	0.855	0.864	0.869	0.883
		10^–7^	0.590	0.602	0.630	0.693	0.854
CL pathway		10^–3^	0.834	0.859	0.862	0.850	0.846
		10^–4^	**0.864**	0.854	0.850	0.859	0.862
		10^–5^	0.765	0.817	0.839	0.855	0.857
		10^–6^	0.699	0.730	0.753	0.769	0.834
		10^–7^	0.654	0.656	0.681	0.688	0.735

a The learning rate and epoch were verified only for seed = 1. The best validation score is shown in bold. LD_50_, 50% lethal dose; BBBP, blood–brain barrier penetration; CL, clearance.

**Table 2:** External Test Results for LD_50_, BBBP, and the CL Pathway^a^

		*n*	AUC	BAC	ACC	sensitivity	specificity	MCC	*F*-measure	precision	recall
LD_50_	DeepSnap-DL method	2894	0.887	0.818	0.779	0.772	0.863	0.393	0.865	0.984	0.772
	MD-based method	2894	0.931	0.805	0.938	0.965	0.645	0.605	0.966	0.967	0.965
	ensemble model	2894	0.942	0.842	0.916	0.931	0.752	0.572	0.953	0.976	0.931
	consensus model	2232–2342		0.916	0.958	0.965	0.866	0.743	0.977	0.989	0.965
BBBP	DeepSnap-DL method	409	0.893	0.824	0.829	0.815	0.834	0.590	0.692	0.601	0.815
	MD-based method	409	0.919	0.849	0.883	0.785	0.913	0.683	0.759	0.735	0.785
	ensemble model	409	0.936	0.853	0.885	0.794	0.912	0.689	0.763	0.736	0.794
	consensus model	328–351		0.918	0.926	0.905	0.932	0.792	0.836	0.776	0.905
CL pathway	DeepSnap-DL method	127	0.883	0.763	0.798	0.863	0.663	0.534	0.853	0.843	0.863
	MD-based method	127	0.900	0.807	0.825	0.858	0.756	0.609	0.869	0.883	0.858
	ensemble model	127	0.908	0.832	0.841	0.858	0.805	0.649	0.880	0.903	0.858
	consensus model	100–110		0.847	0.875	0.915	0.779	0.699	0.912	0.910	0.915

a The mean results are presented for seed = 1–5. LD_50_, 50% lethal dose; BBBP, blood–brain barrier penetration; CL, clearance; DL, deep learning; MD, molecular descriptor; AUC, area under the receiver operating characteristic curve; BAC, balanced accuracy; ACC, accuracy; MCC, Matthews’ correlation coefficient.

### Construction of MD-based Models Using DataRobot for LD_50_, BBBP, and the CL Pathway

First, according to the results of logloss of internal validation, the algorithm was selected, and 100 MDs were selected among 6554 MDs using permutation importance (Tables S4–S6). Second, to build a final predictive model, these 100 MDs were utilized. A different algorithm was selected for each model (Table S7). The results of the evaluation metrics for the test data sets are presented in Table 2. The AUC and BAC were 0.931 and 0.805, respectively, for LD_50_, 0.919 and 0.849, respectively, for BBBP, and 0.900 and 0.807, respectively, for the CL pathway. The results for LD_50_, BBBP, and the CL pathway at each seed are shown in Tables S1–S3.

### Ensemble and Consensus Models Based on the Combined DeepSnap-DL and MD-based Methods

The averages of the predicted probabilities obtained using the DeepSnap-DL and MD-based methods were calculated for the ensemble model. The results of the evaluation metrics of the test sets using the ensemble model are presented in Table 2. The AUC and BAC were, respectively, 0.942 and 0.842 for LD_50_, 0.936 and 0.853 for BBBP, and 0.908 and 0.832 for the CL pathway. The evaluation metrics of the ensemble model were better than those of the DeepSnap-DL and MD-based methods alone. A consensus model was constructed using the results for agreement between the DeepSnap-DL and MD-based methods. Table 2 presents the results of the evaluation metrics of test sets for the consensus model. BAC was 0.916 for LD_50_, 0.918 for BBBP, and 0.847 for the CL pathway. The evaluation metrics of the consensus model were better than those of the ensemble model. However, all compounds were evaluable for the ensemble model, but the number of evaluable compounds was reduced because discordant compounds were removed for the consensus model. Therefore, the number of evaluable compounds was decreased from 2894 to 2232–2342 for LD_50_, from 409 to 328–351 for BBBP, and from 127 to 100–110 for the CL pathway. The results for LD_50_, BBBP, and the CL pathway at each seed are shown in Tables S1–S3.

## Discussion

The present study examined the hyperparameters of the DeepSnap-DL method for LD_50_, BBBP, and the CL pathway. In a previously reported prediction of rat CL, regarding the conditions of the DeepSnap-DL method, we explored hyperparameters with the following conditions: angle of image capture, 65–145° (four conditions); learning rate, 10^–7^ to 10^–3^ (five conditions); and max epoch (five conditions); thus, 100 total conditions were used. Although conditions that result in high predictive performance using the DeepSnap-DL method can be explored by examining this wide range of conditions, it is difficult to apply this approach to drug discovery screening, which has many evaluation targets. Therefore, in the present investigation, based on the previous report of a rat CL predictive model,^15^ we fixed the angle at which images would be captured at 145° and explored 25 conditions, that is, learning rate of 10^–7^ to 10^–3^ (five conditions) and max epoch of 15–300 (five conditions), for LD_50_, BBBP, and the CL pathway. The AUC of DeepSnap (validation) peaked using a learning rate of 10^–5^ and max epoch of 60 for LD_50_, using a learning rate of 10^–4^ and max epoch of 300 for BBBP, and using a learning rate of 10^–4^ and max epoch of 15 for the CL pathway (Table 1). Considering the previous examination results for rat CL (learning rate of 10^–6^ and max epoch of 300), conditions that provide good results with the DeepSnap-DL method can be explored by examining three conditions for the learning rate (10^–6^ to 10^–4^) and five conditions for the max epoch (15–300), giving 15 conditions in total. Thus, we assumed that this approach could be applied to many prediction targets at the drug discovery stage.

The calculation of LD_50_ requires animal experiments.^11^ In recent years, alternative methods have been explored in terms of costs of experiments and ethics. Among them, in silico prediction is attracting attention, and multiple predictive models based on QSAR analysis have been reported.^21,22^ In particular, the collaborative acute toxicity modeling suite (CATMoS) was used to construct a large-scale predictive model for LD_50_.^11^ In the present investigation, we focused on a binary model with the criterion of 50 mg/kg using data sets in the CATMoS in which structural information of compounds was included; predictive models were constructed using the same data set (training set and test set). In this construction of predictive models using a criterion of 50 mg/kg, 32 different organizations constructed predictive models separately, and then a consensus model was constructed by a weighted majority rule based on scores calculated using the predictive model of each group.^11^ Moreover, a predictive model based on the weight-of-evidence approach using the results of five different independent endpoints was also constructed.^11^ It has been reported that the BAC of the consensus model constructed by a weighted majority rule was 0.87 and that of the predictive model constructed by the weight-of-evidence approach was 0.84.^11^ Here, we constructed predictive models using the same data set (training set and test set), which are equally divided chemical space (Figure 1), and obtained a BAC of 0.818 for the DeepSnap-DL method and 0.805 for the MD-based method (Table 2). These values were lower than those obtained using CATMoS (0.84–0.87). Meanwhile, we previously reported approaches for improving predictive performance using an ensemble model and a consensus model combining the DeepSnap-DL and MD-based methods.^15^ In the present study, we investigated this approach to improving predictive performance by combining the DeepSnap-DL and MD-based methods. The BAC was 0.842 with the ensemble model versus 0.916 with the consensus model (Table 2). It is not easy to construct a predictive model by combining models obtained from 32 different organizations such as the construction of a predictive model using CATMoS. However, the ensemble model constructed in the present investigation had similar predictive performance to the model created using CATMoS. Furthermore, the consensus model had the highest BAC of 0.916, although the number of evaluable compounds was decreased from 2894 to 2232–2342 (Table 2). As features of compounds, CATMoS uses MDs or fingerprints. We surmised that reasons for the improved predictive performance for LD_50_ in the present study were that we used images of compounds, which CATMoS does not use, as new features, and we combined the use of images with a method based on MDs.

Compounds that exert their effects on the central nervous system must pass through the BBB to reach their target sites in the brain.^23,24^ Therefore, when developing such compounds, evaluation of their distribution to the central nervous system is essential. However, evaluation of drug distribution to the central nervous system requires in vivo animal experiments. From perspectives of reducing animal experiments and experimental costs, predictive models for distribution to the central nervous system based on QSAR analysis are attracting attention, and many predictive models have been reported.^18,25−27^ In the present study, a combination of DeepSnap-DL and MD-based methods was examined using a BBBP data set of 2049 compounds that was published by Wu et al.,^25^ Chen et al.,^28^ and Martins et al.^18^ Using this data set, Wu et al. and Chen et al. constructed a predictive model with a scaffold split, and Martins et al. narrowed compounds to those with a molecular weight of 600 or lower and then constructed a predictive model using a random split.^18,25^ The reported AUCs of the predictive model developed by Wu et al. and Chen et al. were 0.729 and 0.763, respectively, and the Matthews’ correlation coefficient (MCC) of the predictive model developed by Martins et al. was 0.737. However, the calculated AUC and MCC of the ensemble model constructed by combining the DeepSnap-DL and MD-based methods in the present study were 0.936 and 0.689, respectively (Table 2). Because duplicated compounds in published data sets were removed from the data set used in the present study, our method of dividing the data set into training and test sets, which are equally divided chemical space (Figure 1), differed from those of previous reports. Thus, the results obtained under different conditions were compared. The predictive model constructed in the present study had a higher AUC than that reported by Wu et al. (AUC = 0.729) and Chen et al. (AUC = 0.763). Contrary to this, the predictive model had a lower MCC than that reported by Martins et al. (MCC = 0.737). However, the consensus model developed by combining the DeepSnap-DL and MD-based methods had a greatly improved MCC, which was higher than that reported by Martins et al., although the number of evaluable compounds was reduced from 409 to 328–351 (Table 2). Martins et al. constructed a predictive model by limiting the molecular weight of compounds to 600 or lower and setting a priori probabilities on the basis of Bayesian statistics, according to findings that only 2% of small molecules can cross the BBB.^18^ However, such a setting according to a priori knowledge is not necessarily possible for all evaluation targets. The predictive model combining the DeepSnap-DL and MD-based methods constructed in the present investigation achieved similar or better predictive performance without using such a priori knowledge. Thus, we surmise that this approach can be applied to many evaluation targets because it does not require a priori knowledge.

In the present study, we calculated importance of MDs in the MD-based method for BBBP prediction. The resultant top 10 MDs in terms of importance are presented in Table S8. MDs associated with charge (such as FUnion) and an MD associated with lipophilicity (such as ALOGP) were selected. Because it has been reported that the distribution of compounds to the central nervous system involves the transporter P-glycoprotein,^23,24^ the effect of P-glycoprotein on BBBP was surmised. Ohashi et al. reported a predictive model for transport activity in cells expressing P-glycoprotein.^20^ It has been reported that in the construction of this predictive model, MDs associated with charge such as h_pavgQ and those associated with lipophilicity such as GCUT_SLOGP_0 and GCUT_SLOGP_3 have high degrees of importance. As in previous reports, these MDs associated with charge and lipophilicity had high degrees of importance in the predictive model for BBBP constructed in the present investigation. Moreover, our predictive model also contained PEoED___3D, an MD associated with P-glycoprotein reported by Seelig.^29^ Based on these results, we surmised that BBBP is affected by the transport activity of P-glycoprotein. This predictive model only uses structural information. However, a predictive model combining pieces of information associated with transporter activity other than structural information has also been reported.^30^ Therefore, the predictive performance for BBBP is expected to be further improved by introducing information associated with transporters such as P-glycoprotein as well as combining the DeepSnap-DL and MD-based methods.

The human CL pathway is an important parameter when selecting methods for the prediction of human in vivo CL. Human CL pathways are classified mainly into hepatic metabolism and renal elimination. For compounds that are hepatically metabolized, it has been reported that CL prediction by an in vitro–in vivo correlation using in vitro liver microsomal metabolism test results or two-species allometric scaling in rats and dogs is useful.^19,31^ On the contrary, for compounds that are renally eliminated, it has been reported that approaches such as single-species scaling using monkeys display good predictive accuracy.^19^ Thus, depending on the CL pathways, the most appropriate prediction method for human CL could be different, and it is desirable to know the CL pathway at the drug discovery stage. By knowing the CL pathway, it also becomes possible to surmise drug–drug interactions.^32^ For the prediction of CL pathways, the extended clearance classification system using experimental values of membrane permeability has been reported, although experimental values are needed to perform this prediction.^33^ However, predictions of the CL pathway using only the structural information of compounds have been reported by Kaboudi and Shayanfar and by Lombardo et al.^10,34^ Kaboudi and Shayanfar divided compounds randomly and then constructed predictive models using MDs. The calculated AUC of the predictive models was in the range of 0.776–0.870, and the calculated accuracy (ACC) was in the range of 0.72–0.77.^10^ Lombardo et al. constructed a predictive model using MDs with an ACC of 0.84, although the compounds used differed from those used in the present investigation.^34^ In the present study, we used a data set of 636 compounds created by Kaboudi and Shayanfar based on a report by Lombardo, which are equally divided chemical space (Figure 1), to investigate the predictive models combining the DeepSnap-DL and MD-based methods. The calculated AUC for the ensemble model was 0.908, and the calculated ACC was 0.841. Although a direct comparison is difficult because the Kaboudi and Shayanfar data set does not include labeled training and test sets, our results exceed those of the model developed by Kaboudi and Shayanfar (Table 2). Moreover, the consensus model combining the DeepSnap-DL and MD-based methods had a greatly improved ACC of 0.875 compared with the ensemble model, although the number of evaluable compounds was reduced from 127 to 100–110. Although it is difficult to directly compare performance because the compounds utilized to construct the models differ, the present study had better performance than that reported by Lombardo (ACC = 0.841). The report by Kaboudi and Shayanfar and that by Lombardo used MDs or information on compound fragments. We surmised that in the present study, the predictive performance was greatly improved because images of compounds were incorporated as new features into the predictive models in addition to these features. Lombardo et al. also reported a predictive model for the CL pathway that used only a group of compounds excreted through the liver or kidney alone, with the excretion rate of 70% or higher. In this predictive model, the ACC was increased to 0.88 by limiting compounds used in the model.^34^ In the present investigation and that reported by Kaboudi and Shayanfar, such limitation of compounds based on the proportion of excretion was not performed. An improvement in predictive performance is expected by both constructing predictive models combining the DeepSnap-DL and MD-based methods and developing strategies such as limiting compounds to those with high excretion rates.

Although the consensus model has been shown to have the highest prediction accuracy for each evaluation target, it is not possible to evaluate all test compounds. In fact, the number of compounds that can be evaluated by the consensus model has been reduced (Table 2). In the early stages of drug screening, many compounds need to be evaluated comprehensively without omission. Therefore, practical use can be achieved by first evaluating compounds using the consensus model and then using the ensemble model for compounds that cannot be evaluated.

## Conclusions

This study investigated the application of ensemble and consensus models combining the DeepSnap-DL and MD-based methods to new prediction targets. For LD_50_, BBBP, and the CL pathway, which are expected to be applied as targets for models in drug discovery, an improvement in predictive performance was observed using ensemble and consensus models. This approach does not require the construction of complex predictive models. This strategy enables the easy construction of high-performance predictive models by combining two predictive models. This combination QSAR method enables virtual screening in a library of compounds, and it is expected to accelerate the drug discovery. Moreover, further improvement in predictive performance is expected by combining an approach based on a priori knowledge and limiting the compounds used for constructing predictive models based on knowledge about each evaluation target.

## Materials and Methods

### Experimental Data

To construct the predictive model for LD_50_, we selected a “very toxic” data set with a threshold of LD_50_ ≤ 50 mg/kg based on a report by Mansouri et al.^11^ In the present investigation, we used the same training and test sets as those of previous reports consisting of 11,886 compounds to construct predictive models (Tables 3 and S9).

**Table 3:** Number of Chemical Compounds in the Training and Test Data Sets^a^

	score	training	test	sum
LD_50_	TRUE	741	243	984
	FALSE	8251	2651	10,902
	sum	8992	2894	11,886
BBBP	0	386	96	482
	1	1254	313	1567
	sum	1640	409	2049
CL pathway	hepatic metabolism	345	86	431
	renal elimination	164	41	205
	sum	509	127	636

a BBBP = 0, log BB < −1; BBBP = 1, log BB ≥ −1.

The verification of BBBP was performed using the same data set reported by Wu et al.^25^ This data set is a binary classification data set consisting of “0” compounds with poor distribution to the central nervous system, that is, blood–brain partition (log BB) < −1, and “1” compounds with good distribution, that is, log BB ≥ −1. We obtained data for 2050 compounds from the website https://moleculenet.org/datasets-1. Among these compounds, BRL53080 and loperamide had the same structure, and loperamide was removed, resulting in a data set of 2049 compounds (Tables 3 and S10). In the present investigation, data were sorted by objective variables and then randomly divided into training and test sets at a 4:1 ratio.

For the CL pathway, we obtained the same data set of 636 compounds reported by Kaboudi and Shayanfar (Tables 3 and S11).^10^ As in their report, the compounds were sorted by objective variables (hepatic metabolism and renal elimination) and log *D* and then randomly divided into training and test sets at a 4:1 ratio.

### Calculation of MDs

Among the structural data of compounds, those containing counterions and water molecules were removed from the data sets by processing the disposal salts using Molecular Operating Environment (MOE) version 2019.01 (MOLSIS Inc., Tokyo, Japan). An RDKit was applied to optimize the three-dimensional (3D) structure of each compound for LD_50_, BBBP, and the CL pathway using MMFF as the force field based on the previous report,^35^ respectively. Furthermore, MOE, alvaDesc (2.0.2) (Alvascience srl, Lecco, Italy), and ADMET Predictor (9.5.0.16) (SimulationsPlus, Lancaster, CA, USA) were utilized to calculate MDs. Any descriptors of string type were removed in ADMET Predictor when MDs were generated. Overall, 6554 descriptors were selected for further analysis.

### Splitting of Data Sets into Training and Test Sets and Their Verification by Chemical Space Analysis

Because training and test sets were specified in previous reports for LD_50_, the same data set from a previous report was used.^11^ The compounds for BBBP and the CL pathway were randomly divided into training and test sets at a ratio of 4:1 after applying stratified random sampling. Eleven molecular parameters were used to investigate the applicability domain by utilizing the PCA with JMP Pro software 14.3.0 (SAS Institute Inc., Cary, NC, USA).^20^ The parameters evaluated in this study were molecular weight, Slog *P* (log octanol/water partition coefficient), topological polar surface area, h_logD (octanol/water distribution coefficient, pH = 7), h_pKa (acidity, pH = 7), h_pKb (basicity, pH = 7), a_acc (number of H-bond acceptor atoms), a_aro (number of aromatic atoms), a_don (number of H-bond donor atoms), b_ar (number of aromatic bonds), and b_rotN (number of rotatable bonds). We calculated three PCs (PC1–3).

### DeepSnap

The Java viewer software Jmol was used to depict 3D chemical structures as 3D ball-and-stick models in different colors for each atom (Figure S1).^14,16,17,36−39^ In this study, the 3D chemical structures were automatically captured as snapshots at 145° for three axes (*x*-, *y*-, and *z*-axes). Other parameters used in this study for the DeepSnap depiction process were as follows: 256 × 256 image pixel (RGB), 100 molecules/SDF file, zoom factor 100%, atom size of 23% for the van der Waals radius, bond radius of 15 mÅ, minimum bond distance of 0.4, and bond tolerance of 0.8.:^14,16,17,36−39^ after sorting the training data sets based on the target variable, they were randomly divided into the DeepSnap (training) and DeepSnap (validation) sets at a ratio of 3:1 for BBBP, LD_50_, and the CL pathway. The data sets for DeepSnap-DL consists of DeepSnap (training), DeepSnap (validation), and test sets (Figure S2).

### Deep Learning

Snapshots of two-dimensional images produced by DeepSnap were saved as PNG files and resized using NVIDIA DL GPU Training System (DIGITS) version 6.0.0 software (NVIDIA, Santa Clara, CA, USA) on the Tesla-V100 four-GPU system (32 GB).^14,16,17,36−39^ We used pre-trained DL model Caffe^40^ to quickly train and fine-tune the highly accurate convolutional neural network (CNN) and software on the ubuntu distribution 16.04LTS. GoogLeNet and Adam were used for the deep CNN architecture and optimization, respectively. In the DeepSnap-DL method, the predictive models were constructed by DeepSnap (training) data sets using 15–300 epochs with one snapshot interval and one validation interval in each epoch, one random seed, a learning rate of 10^–7^ to 10^–3^, and default conditions for the batch size, batch accumulation, policy, step size, and gamma in DIGITS. The lowest loss value in the DeepSnap (validation) data sets represented the error rate in the results obtained in the DeepSnap (validation) data sets and the corresponding labeled data set, and this condition was used to evaluate the prediction in the test set. The probability for each image of one molecule captured at different angles (*x*-, *y*-, and *z*-axes) using the DeepSnap-DL method was calculated with the lowest loss (DeepSnap [validation]) conditions. The medians of all predicted values were used as the representative values for target molecules.^14,16,17,36−39^ To construct the predictive model with random seed values of 2–4, the predictive model was constructed using the learning rate and epoch determined with a random seed value of 1.

### Construction of Predictive Models Based on MDs

**Figure 2:**
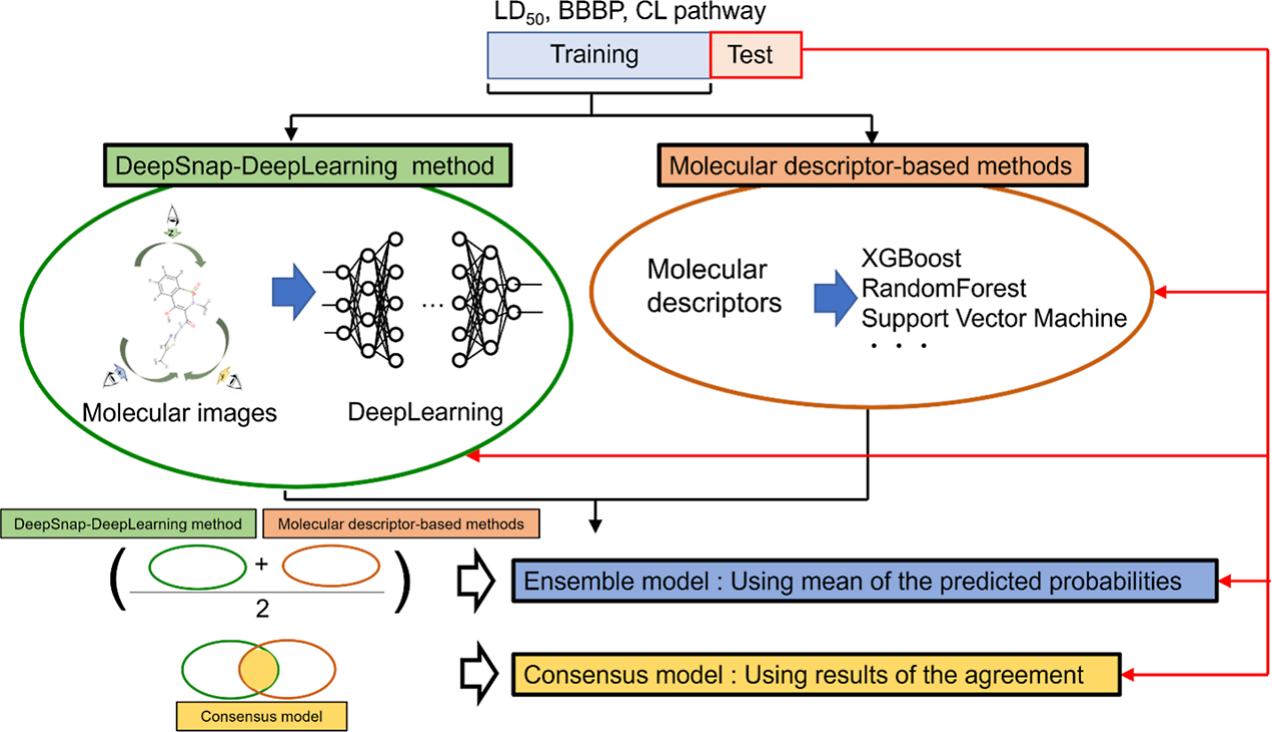
Flowchart of the modeling process for LD_50_, BBBP, and the CL pathway. The training data set was utilized to construct predictive models by using the MD-based method by DataRobot and the DeepSnap-DL method. Ensemble and consensus models were constructed by combining the MD-based and DeepSnap-DL methods. The evaluation metrics of each predictive model were calculated using the test sets. LD_50_, 50% lethal dose; BBBP, blood–brain barrier penetration; CL, clearance, DL, deep learning.

Model construction and analysis were performed using DataRobot (SaaS, DataRobot, Tokyo, Japan) from May 20, 2022 to November 2, 2022. DataRobot automatically performed a modeling competition with a wide range of selection of algorithm and data preprocessing techniques, as reported previously.^41,42^ Five-fold cross-validation was implemented, and other conditions were as described previously.^15^ After selecting the models based on the logloss scores of internal validation, we selected 100 MDs from 6554 candidate MDs using permutation importance. Fourteen-forty-two models were constructed and algorithms were selected on the basis of the validation results as the final algorithm (Table S7). After the logloss scores of internal validation, we constructed the best model using 100% of the training data. The final model was utilized to calculate the predictive performance of the test sets (Figure 2).

### Combined DeepSnap-DL and MD-based Methods

The combined use of the DeepSnap-DL and MD-based methods was investigated in this study. In the first method, the predictive probabilities obtained by the methods were averaged, and these values were used as the predictive probabilities of the new predictive model (ensemble model, Figure 2). In the second method, the results that agreed between the two methods were adopted (consensus model, Figure 2).

### Evaluation of the Models

The performance of each model in predicting LD_50_, BBBP, and the CL pathway was evaluated for the following metrics: AUC, ACC, BAC, sensitivity, specificity, F-measure precision, recall, and MCC. The metrics were calculated using KNIME (4.3.4) (KNIME, Konstanz, Germany) and defined as follows
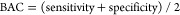
where ACC = (TP + TN)/(TP + FP + TN + FN)
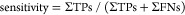

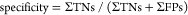

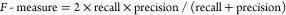
where precision = TP/(TP + FP) and recall = TP/(TP + FN); and
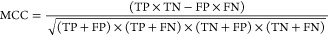
where TP, FN, TN, and FP denote true positive, false negative, true negative, and false positive, respectively. To determine the optimal cutoffs for true positive, false negative, true negative, and false positive, the method for maximizing sensitivity (1 – specificity), termed the Youden index,^43,44^ was applied.

## Supporting Information Available

DeepSnap procedure And split pattern for DeepSnap-DL (PDF)
External test results for the CL pathway (seed = 1–5); external test results for BBBP (seed = 1–5); external test results for LD_50_ (seed = 1–5); top three validation results for the LD_50_ prediction model using 100 descriptors; top three validation results for the BBBP prediction model using 100 descriptors; top three validation results for the CL pathway prediction model using 100 descriptors; final algorithm selected by DataRobot using 100 descriptors; top 10 ranked descriptors in BBBP predictive models using an MD-based method; list of studied compounds for LD_50_; list of studied compounds for BBBP; and list of studied compounds for the CL pathway (XLSX)

## Notes

Data for all compounds are included in the Supporting Information. Commercial software including MOE (https://www.chemcomp.com/index.htm) alvaDesc (https://www.alvascience.com/alvadesc/), ADMET Predictor (https://www.simulations-plus.com/software/admetpredictor/), JMP Pro software (https://www.jmp.com/en_us/home.html), DataRobot (https://www.datarobot.com/jp/), and DIGITS (https://developer.nvidia.com/digits) were used. Open-source software including KNIME (https://www.knime.com/) was used. DeepSnap is written in Python 3 and code can be provided if needed.

## Abbreviations

QSAR: quantitative structure–activity relationship
MDs: molecular descriptors
DL: deep learning
LD_50_: 50% lethal dose
CL: clearance
BBBP: blood–brain barrier penetration
BBB: blood–brain barrier
PCA: principal component analysis
PC: principal component
AUC: area under the curve
BAC: balanced accuracy
CATMoS: collaborative acute toxicity modeling suite
MCC: Matthews’ correlation coefficient
ACC: accuracy
log BB: blood–brain partition
MOE: Molecular Operating Environment
3D: three-dimensional
Slog *P*: log octanol/water partition coefficient
h_logD: octanol/water distribution coefficient
h_pKa: acidity
h_pKb: basicity
a_acc: number of H-bond acceptor atoms
a_aro: number of aromatic atoms
a_don: number of H-bond donor atoms
b_ar: number of aromatic bonds
b_rotN: number of rotatable bonds
DIGITS: NVIDIA DL GPU Training System
CNN: convolutional neural network
