# Supplementary material for: Predictive Models Based on Molecular Images and Molecular Descriptors for Drug Screening
Source: ACS Omega. 2023 Sep 13;8(40):37186–95. doi: 10.1021/acsomega.3c04073 (PMC10568689; doi:10.1021/acsomega.3c04073)
Supplement: Supplementary file 1 — ao3c04073_si_001.pdf [file ao3c04073_si_001.pdf]

Predictive models based on molecular images and molecular descriptors for drug screening

*Hideaki Mamada[1], Mari Takahashi[1], Mizuki Ogino[1], Yukihiro Nomura[1], Yoshihiro Uesawa[*2]*

[1] Drug Metabolism and Pharmacokinetics Research Laboratories, Central Pharmaceutical Research Institute, Japan Tobacco Inc., 1-1 Murasaki-cho, Takatsuki, Osaka 569-1125, Japan.

[2] Department of Medical Molecular Informatics, Meiji Pharmaceutical University, 2-522-1 Noshio, Kiyose, Tokyo 204-858, Japan

[*]Corresponding author.

Tel.: +81-42-495-8983; Fax: +81-42-495-8983;

E-mail address: uesawa@my-pharm.ac.jp (Y.U.)

Supporting Information 1

Supporting Tables (file type: Excel)

Table S1. External test results for the CL pathway (seed = 1–5)

Table S2. External test results for BBBP (seed = 1–5)

Table S3. External test results for LD50 (seed = 1–5)

Table S4. Top three validation results for the LD50 prediction model using 100 descriptors

Table S5. Top three validation results for the BBBP prediction model using 100 descriptors

Table S6. Top three validation results for the CL pathway prediction model using 100 descriptors

Table S7. Final algorithm selected by DataRobot using 100 descriptors

Table S8. Top 10 ranked descriptors in BBBP predictive models using a molecular descriptor-based method

Table S9. List of studied compounds for LD_{50}

Table S10. List of studied compounds for BBBP

Table S11. List of studied compounds for the CL pathway

Supporting Information 2

Supporting Figures (file type; WORD)

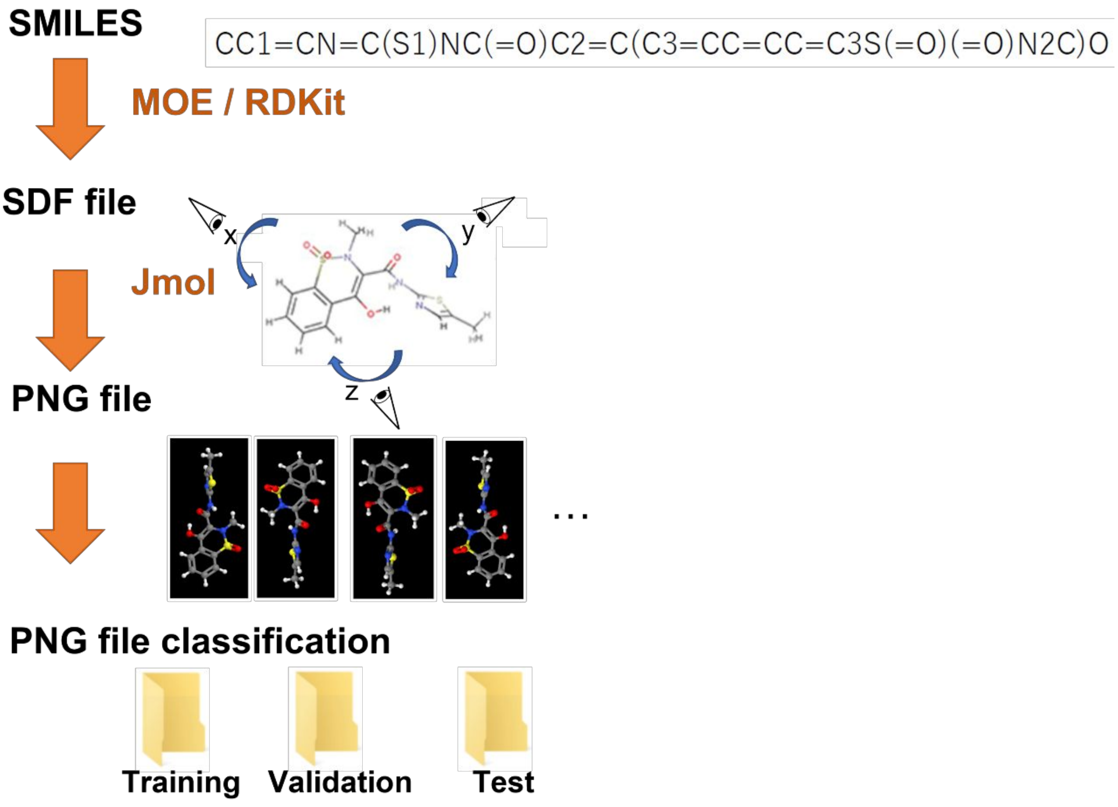

Figure S1. The Deep Snap procedure

The chemical structure of the SMILES format is converted 3D chemical structure of SDF file. Deep-Snap was used to obtain images of the compounds from various angles as PNG

files. The compounds were then split into training, validation, and test sets.

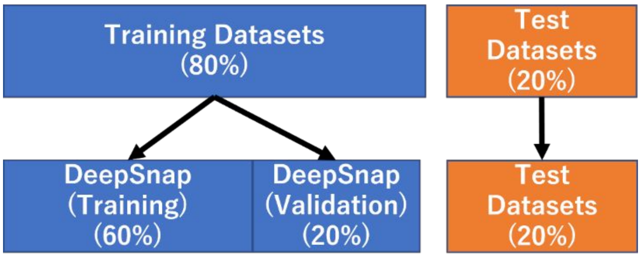

Figure S2. Split pattern for DeepSnap-DL

The training datasets were randomly divided into the DeepSnap (Training) and DeepSnap (Validation) sets at a ratio of 3:1.
